# Postoperative Hardware-Related Infection from *Kytococcus schroeteri*: Its Association with Prosthetic Material and Hematological Malignancies—A Report of a Case and Review of Existing Literature

**DOI:** 10.1155/2019/6936472

**Published:** 2019-03-24

**Authors:** Aditya S. Shah, Prakhar Vijayvargiya, Sarah Jung, John W. Wilson

**Affiliations:** ^1^Mayo Clinic, Department of Infectious Diseases, 200 First Street SW, Rochester, MN 55905, USA; ^2^Mayo Clinic, Department of Clinical Microbiology, 200 First Street SW, Rochester, MN 55905, USA

## Abstract

**Introduction:**

*Kytococcus schroeteri* is an infrequently isolated Gram-positive coccus often encountered as a commensal bacterium. Only eighteen cases of human infection associated with this organism have been previously reported. Most of these cases involved patients with implanted prosthetic materials or patients with immunosuppressive conditions. It has been described in prosthetic valve endocarditis and in select patients with hematologic diseases but only one prior report as being involved in osteoarticular infections.

**Case Presentation:**

We describe a case of postsurgical osteoarticular hardware-related infection by *K. schroeteri* and discuss a possible association with implanted prosthetic material.

**Conclusion:**

Other clinical presentations of *K. schroeteri*, including reported infection syndromes, antimicrobial susceptibility profiles, and treatment outcomes, are also reviewed.

## 1. Introduction

*Kytococcus schroeteri* is a Gram-positive coccus that was first identified by 16S rDNA analysis in 2002 [1]. It is a yellow-pigmented, aerobically growing, nonencapsulated, nonmotile bacterium. The natural habitat of *K. schroeteri* is not well defined, but other members of the genus *Kytococcus* (*K. sedentarius*) have been isolated from human skin. Human infection caused by this commensal bacterium is uncommon. The first case of *K. schroeteri* infection was described in 2002, in a patient with prosthetic valve endocarditis [1]. Over the subsequent sixteen years, 18 cases have been reported. Of those, only one prior case has been reported of *K. schroeteri* causing an osteoarticular infection [2]. We discuss a case of *K. schroeteri* associated with a postsurgical hardware-related infection and review its known role in other select infection syndromes.

## 2. Case Report

Our patient was an 80-year-old female with a history of chronic adrenal insufficiency on oral prednisone. She suffered a left-sided intertrochanteric hip fracture and underwent a surgical implantation of a cephalomedullary nail to stabilize the femoral neck. Over the next two weeks, she developed continuous drainage from the surgical incision. On presentation to the hospital, she had ecchymoses on her left flank and serosanguinous drainage from her left hip incision. She was afebrile on admission but had an elevated white blood cell count of 29 × 10^9^/L. An ultrasound of the hip and groin region showed a hematoma and a large left groin pseudoaneurysm from the profunda femoral artery, which was confirmed by a CT angiogram. The patient underwent coil embolization of the pseudoaneurysm and surgical wound debridement.

### 2.1. Investigations and Diagnosis

There were multiple positive culture results for *K. schroeteri* on hip tissue/peri-joint tissue sent intraoperatively; and the treating infectious disease team with orthopedic infectious disease speciality focus felt this was real and constituted a prosthetic joint infection, warranting full treatment and suppression. This strain was resistant to penicillin but susceptible to clindamycin and vancomycin by Mueller–Hinton agar dilution.

### 2.2. Treatment

The patient was discharged to a care facility and received four weeks of daptomycin. This medication was chosen for out-of-hospital convenience of administration, owing to the once-a-day dosing. She recovered complete mobility of the joint and had no further complications in her course.

## 3. Discussion

The increasing number of case reports, including our own, suggests that *K. schroeteri* can be a formidable pathogen in the appropriate host. The significance of *Kytococcus* as a human pathogen may not have been fully recognized in years past or previously misidentified as *Micrococcus* sp. Recent case reports have been able to identify this organism by molecular sequencing or using matrix-assisted laser desorption/ionization–time-of-flight mass spectrometry (MALDI-TOF MS) technology, as in our case. The organism grew on a blood agar plate (a trypticase soy agar plate with 5% sheep's blood) at 35° Celsius in a CO_2_-enriched environment. Colony growth showed muddy yellow-pigmented bacteria. In this report, we identify a case where *Kytococcus* was isolated from bacterial cultures and was implicated in a postsurgical hardware-related infection.

To our knowledge, this is the second known report of an osteoarticular infection caused by *K. schroeteri*. The first known case of orthopedic infection caused by this organism was described in a case of spondylodiscitis after surgery [2]. Our patient was moderately immunocompromised as she was taking prednisone 10 mg daily for several years for her chronic adrenal insufficiency. Our review of the literature (Table 1) identified 13 other cases (Table 1) with *Kytococcus* infection that were associated with implanted foreign material: eight with prosthetic valve endocarditis, two with VP shunt infections, one with prosthetic discitis, one with silicon tendon graft infection, and one with medullary nail infection (our patient). These reports underscore the importance of evaluating for foreign body infections in patients who have a positive blood culture with *K. schroeteri*.

Our literature review also identified 6 reported cases of *Kytococcus* pneumonia. Five of these were in conjunction with hematological malignancy (4 AML and 1 hairy cell leukemia) [11–13, 17]. The sixth patient had no hematologic condition but was moderately immunocompromised by taking prednisone 20 mg daily for 2 years for management of refractory asthma [3]. Indeed, at our institution, we have also encountered a separate and additional patient with acute myelogenous leukemia who developed fever, hypoxic respiratory failure, and consolidated infiltrates on chest imaging. *K. schroeteri* was identified in multiple cultures from respiratory samples (unpublished).

*K. schroeteri* is frequently misidentified as *Micrococcus* sp. because of similar morphological features. However, a distinction between the two is important since *Micrococcus* sp. is inherently susceptible to penicillin and oxacillin, whereas *Kytococcus* sp. is not. Antimicrobial susceptibility testing (AST) of *K. schroeteri* from both our patients demonstrated in vitro resistance to penicillin and susceptibility to vancomycin and clindamycin. We also reviewed the antimicrobial susceptibility testing (AST) profile from all prior cases associated with this organism (Table 1), and the characteristic resistance to penicillin and susceptibility to vancomycin were uniform.

An important point to highlight is the fact that there are no formal established MIC breakpoints by the Clinical Laboratory Standards Institute (CLSI); therefore, the laboratory interpretation of MIC breakpoints reflecting drug susceptibility vs. resistance are implied and must be interpreted with caution. In 2015, CLSI did publish MIC breakpoints for *Micrococcus* sp. Several genera, including *Kytococcus* sp., have been included under the *Micrococcus* group for AST interpretation. While CLSI defines clinical “susceptible” vs. “resistant” *Micrococcus* MIC breakpoints for penicillin, there is no formal “resistant” MIC breakpoint defined for vancomycin. Rather, only an MIC breakpoint below which vancomycin susceptibility is reported. Therefore, vancomycin resistance is implied when reported at or above the MIC breakpoint. Whether there are clinically relevant MIC breakpoint differences between *Micrococcus* and *Kytococcus* has not been adequately determined at this time. An AST database of *Kytococcus* and *Micrococcus* isolates by matrix-assisted laser desorption ionization-time of flight (MALDI-TOF) has been developed at our center to augment the currently available CLSI reporting standards.

In summary, we describe the second known case of an osteoarticular infection from *K. schroeteri* and reviewed the published reports of *K. schroeteri* disease. While often considered part of the natural skin bacterial flora, human infections with *K. schroeteri* do occur and have presented in patients with infected implanted prosthetic material and as respiratory infections in patients with significant immunosuppressive conditions, including select hematologic malignancies. *Kytococcus* appears to be susceptible to vancomycin and resistant to penicillin when grouped with *Micrococcus* by CLSI, although species-specific MIC breakpoints have not yet been formally established. The advancements made in laboratory diagnostic techniques along with rising applications of prosthetic materials may collectively enable an increased awareness of *Kytococcus* sp. as a potentially formidable human pathogen. Early identification and appropriate treatment will be critical toward successful outcomes.

## Figures and Tables

**Table 1:** Reported cases of *Kytococcus* infection.

Age (years)/Sex	Immunocompromising condition	Primary source of infection	Therapy	Outcome	Report
34/F	None	Prosthetic valve endocarditis	Vancomycin, rifampin, and gentamicin	Recovered	Becker et al. [1]
71/F	Asthma on chronic prednisone therapy	Pneumonia/bacteremia	Ceftriaxone and ofloxacin	Deceased	Mohammedi et al. [3]
73/M	None	Prosthetic valve endocarditis	Teicoplanin, rifampin, and gentamicin	Recovered	Le Brun et al. [4]
49/F	None	Prosthetic valve endocarditis	Vancomycin and gentamicin, followed by pristinamycin and vancomycin	Recovered	Mnif et al. [5]
38/F	None	Prosthetic valve endocarditis	Vancomycin and gentamicin, followed by levofloxacin and rifampin	Recovered	Aepinus et al. [6]
70/M	None	Prosthetic valve endocarditis	Amoxicillin and gentamicin	Recovered	Renvoise et al. [7]
1/M	None	VP shunt	Vancomycin and rifampin	Recovered	Jourdain et al. [8]
72/M	None	Prosthetic valve endocarditis	Vancomycin, rifampin, and gentamicin	Recovered	Poyet et al. [9]
64/M	None	Prosthetic valve endocarditis	Vancomycin, rifampin, and gentamicin	Recovered	Yousri et al. [10]
50/F	Type 2 diabetes	Prosthetic discitis	Ofloxacin and rifampin	Recovered	Jacquier et al. [2]
40/M	Acute myeloid leukemia	Pneumonia/bacteremia	Vancomycin, rifampin, and gentamicin	Deceased	Hodiamont et al. [11]
52/M	Acute myeloid leukemia	Pneumonia/bacteremia	Vancomycin and ceftazidime	Deceased	Hodiamont et al. [11]
43/F	Acute myeloid leukemia	Pneumonia and bacteremia	Vancomycin/piperacillin/tazobactam, vancomycin/meropenem, and linezolid, discharged on TMP/SMX	Recovered	Blennow et al. [12]
68/M	Acute myeloid leukemia	Pneumonia and bacteremia	Vancomycin	Deceased	Nagler et al. [13]
45/M	None	Silicone tendon graft	Oral doxycycline	Recovered	Chan et al. [14]
53/M	None	Prosthetic valve endocarditis	Daptomycin	Recovered	Liu et al. [15]
3/F	Ganglioma	VP shunt	Cefuroxime and gentamicin	Recovered	Schaumburg et al. [16]
51/F	Hairy cell leukemia	Pneumonia and bacteremia	Vancomycin and piperacillin/tazobactam	Deceased	Amaraneni et al. [17]
80/F	None	Medullary nail	Daptomycin	Recovered	Shah et al. 2017

## Abbreviations

MALDI-TOF MS7: Matrix-assisted laser desorption/ionization-time-of-flight mass spectrometry
AST: Antimicrobial susceptibility testing
CLSI: Clinical Laboratory Standards Institute
MIC: Minimum inhibitory concentration
AML: Acute myelogenous leukemia.
